# The Dual Role of Surfactant Protein-D in Vascular Inflammation and Development of Cardiovascular Disease

**DOI:** 10.3389/fimmu.2019.02264

**Published:** 2019-09-20

**Authors:** Kimmie B. Colmorten, Anders Bathum Nexoe, Grith L. Sorensen

**Affiliations:** Department of Molecular Medicine, Faculty of Health Sciences, University of Southern Denmark, Odense, Denmark

**Keywords:** collectins, SP-D, cardiovascular disease, atherosclerosis, inflammation

## Abstract

Cardiovascular disease (CVD) is responsible for 31% of all global deaths. Atherosclerosis is the major cause of cardiovascular disease and is a chronic inflammatory disorder in the arteries. Atherosclerosis is characterized by the accumulation of cholesterol, extracellular matrix, and immune cells in the vascular wall. Recently, the collectin surfactant protein-D (SP-D), an important regulator of the pulmonary immune response, was found to be expressed in the vasculature. Several *in vitro* studies have examined the role of SP-D in the vascular inflammation leading to atherosclerosis. These studies show that SP-D plays a dual role in the development of atherosclerosis. In general, SP-D shows anti-inflammatory properties, and dampens local inflammation in the vessel, as well as systemic inflammation. However, SP-D can also exert a pro-inflammatory role, as it stimulates C-C chemokine receptor 2 inflammatory blood monocytes to secrete tumor necrosis-factor α and increases secretion of interferon-γ from natural killer cells. *In vivo* studies examining the role of SP-D in the development of atherosclerosis agree that SP-D plays a proatherogenic role, with SP-D knockout mice having smaller atherosclerotic plaque areas, which might be caused by a decreased systemic inflammation. Clinical studies examining the association between SP-D and cardiovascular disease have reported a positive association between circulatory SP-D level, carotid intima-media thickness, and coronary artery calcification. Other studies have found that circulatory SP-D is correlated with increased risk of both total and cardiovascular disease mortality. Both *in vitro, in vivo*, and *clinical studies* examining the relationship between SP-D and CVDs will be discussed in this review.

## Introduction

Cardiovascular disease (CVD) is the most frequent cause of death worldwide, with an estimated 17.9 million deaths every year, accounting for about 31% of all global deaths (1). Atherosclerosis is the major cause of CVD and is caused by both genetic and environmental factors (2, 3). We know that atherosclerosis is a chronic inflammatory disorder of the arteries characterized by the accumulation of cholesterol, extracellular matrix, and immune cells in the subendothelial space. Both the innate and adaptive immune systems are implicated in the development of atherosclerotic plaques (4).

Briefly, circulating lipoprotein particles, such as low-density lipoprotein (LDL), can penetrate the arterial wall and accumulate in the subendothelial space. Atherogenic modification of LDL, such as oxidation (oxLDL), induces an immune response triggering immune cells, including neutrophils, natural killer (NK) cells, and monocytes/macrophages, which are the first responders. Macrophages especially participate in the engulfment and accumulation of intracellular oxLDL, leading to the transition of well-functioning macrophages to lipid-filled foam cells (5, 6). Activation of the innate immune cells leads to the secretion of various pro-inflammatory cytokines, where interleukin (IL)-1 (7–9), interferon (IFN)-γ (10–12), and tumor necrosis factor (TNF-)α (13, 14) have an especially proatherogenic effect (6). IL-6 is also a major contributor to the development of atherosclerosis by playing a dual pathological and protective role (15–17).

Following activation of an innate immune response, an adaptive immune response is triggered and mediated by T- and B-cells. Activation of type 1 T helper (Th1) cells by exogenous or endogenous antigens, including oxLDL, leads to the secretion of IFN-γ and enhancement of monocyte infiltration, foam-cell formation, lipid accumulation, and macrophage activation (4).

Recently, the collectin surfactant protein (SP)-D, an important regulator of the innate immune response in the lung, was shown to be expressed in the vasculature, where it regulates local inflammation (18, 19).

This review gives a comprehensive overview of recent *in vitro, in vivo*, and *clinical studies* that have attempted to examine SP-D-mediated inflammation in the vasculature and its function in the pathogenesis of CVD.

## The Collectin Family and Roles in Inflammation

The collectins are members of a superfamily of collagenous, calcium-dependent (C-type) lectins. All members of the collectin family are characterized by the presence of a cysteine-rich N-terminal non-collagenous domain, a collagen-like domain, an α-helical coiled-coil neck domain, and a globular C-type lectin domain (20). These are soluble effector proteins that also possess the property of pattern recognition by recognizing terminal conserved carbohydrate domains on microbes, leading to complement activation (20). The family includes mannose-binding proteins (MBLs), SP-A, SP-D, and three novel human defense collagens; collectin-10, collectin-11, and collectin-12 (21–23). The collectins are known to be an important part of the innate immune defense due to their interaction with a range of microbes. Binding of MBL leads to opsonization of the microorganisms through complement activation via the lectin pathway, whereas SP-A and SP-D have more direct antimicrobial effects (24). SP-D/-A cooperate with alveolar macrophages to facilitate phagocytosis via binding of bacteria, viruses, fungi, and helminthic parasites for clearance through opsonization (24). The collectins do not only exert anti-microbial effects but possess dual biological activity either by suppressing or enhancing the production of pro-inflammatory cytokines (25, 26). Gardai et al. (25) suggested that collectins are capable of differential cellular receptor binding through either their collagen-like domain or their C-type lectin domain, and thereby initiate pro-inflammatory or anti-inflammatory signaling, respectively. Under normal conditions, collectins bind the cellular receptor, signal regulatory protein (SIRP)-α, on innate immune cells via their C-type lectin domain and prevent pro-inflammatory signaling. When pathogens are present, the C-type lectin domain is occupied by its corresponding ligand, and the collectins will then interact with the innate immune cells through a receptor complex of cluster of differentiation (CD)91 and calreticulin. This binding is mediated through the collagen-like domain on the collectin and induces pro-inflammatory signaling (25). Gardai et al. (25) only validated the hypothesis for SP-A, but a study by Fournier et al. validated the binding between SP-D and SIRP-α (27). Moreover, a series of additional cellular receptors have been suggested as mediators of SP-D signaling. Central receptors are mentioned beneath.

## The Structure of SP-D

**Figure 1:**
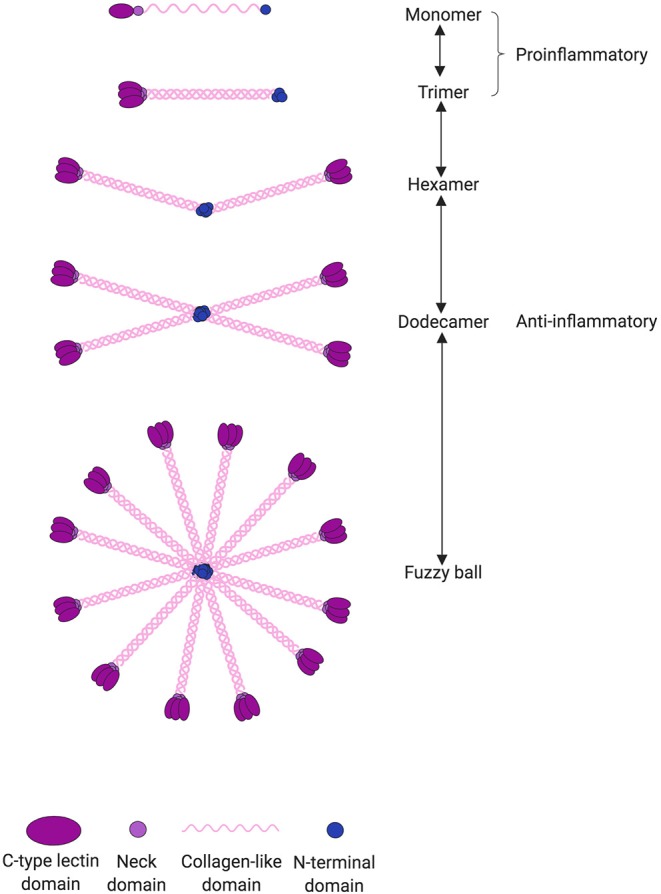
SP-D can undergo several post-translational modifications leading to changes in both structure and function. SP-D is capable of multimerization depending on the local environment. The most common isoform is the dodecameric structure, which is thought to have anti-inflammatory properties through binding via its C-type lectin domain. However, in an inflammatory or more acidic environment, the dodecameric form can change to a trimeric or monomeric isoform. The monomers and trimers are believed to have pro-inflammatory properties through binding via its collagen-like domain. When pathogens are present, the C-type lectin domain is occupied by its corresponding ligand and thus incapable of eliciting its anti-inflammatory signaling.

SP-D is a large oligomeric structure, composed of oligomers of 130 kDa subunits (28). The subunits are comprised of three identical polypeptide chains containing an N-linked oligosaccharide structure (Figure 1). Following assembly of the triple-helical collagen-like domain, the N-terminal cysteine residues form disulfide bridges between monomers, dictating the degree of multimerization and thus the SP-D isoform (28). Human SP-D has different isoforms; the 520 kDa dodecameric isoform structure is the most commonly found in bronchoalveolar lavage fluid (BALF), but also fuzzy ball, trimeric, dimeric, and monomeric isoforms are present in BALF from healthy subjects (29). To understand the function of SP-D, it is important to keep in mind that a shift in SP-D isoforms can lead to changes in its pro- or anti-inflammatory role. The importance of SP-D isoforms will be discussed in this review.

## SP-D Sites of Expression and Function

For many years, SP-D/-A were referred to as lung-specific; they were discovered in the phospholipid-rich pulmonary surfactant (28, 30). The fact that surfactant-like lubrication is produced and secreted by a number of other organs led to the hypothesis that SP-D/-A are also synthesized in extra-pulmonary tissues.

SP-D/-A are primarily synthesized in the lung, where they are produced and secreted onto the epithelial surface by alveolar type II epithelial cells and unciliated bronchial epithelial cells (31). In the lung, SP-D/-A function as regulators of lipid levels in the pulmonary surfactant, where they also exert their antimicrobial effects (24, 32). Since SP-D/-A were first discovered in the lung, this is also the organ where their function is most extensively examined. Studies on SP-D/-A knockout (KO) mice generally reported increased susceptibility to acute and chronic infections and a general enhanced acute inflammatory response upon pathogen or smoke exposure of the lung (33–36). Taken together, these studies underline the important roles of SP-D/-A in the pulmonary innate host defense and regulation of surfactant homeostasis (31).

Even though the primary site of SP-D expression is in the lung, it has been localized to a range of non-pulmonary tissues, including the external or luminal surfaces of the gastrointestinal tract (37, 38), glandular system (37, 38), reproductive tract (37–39), urinary tract (37, 38), and in the cardiovascular (CV) system (18, 19). In the CV system, SP-D expression has been localized to endothelial and smooth muscle cells (SMCs), where its suggested function is the modulation of inflammatory signaling (18, 19). The sources of circulating SP-D are not fully clarified. In addition to lung spillover as primary contributor, it is possible that SP-D can be secreted from the arterial wall and affect the total serum concentration. Recent studies by Kati et al. and Gargiulo et al. have shown that circulatory SP-D levels correlate with the presence of pulmonary embolism (40) and with alveolar leakage in heart failure (41), thereby supporting the hypothesis that circulatory SP-D variation is partly a consequence of CVD-mediated lung damage. Based on recent clinical studies pinpointing SP-D as a systemic biomarker of CVD morbidity and mortality (42, 43), it is suggested that SP-D has an important function in the cardiovascular system as a regulator of inflammation, which might have implications for atherosclerosis and CVD.

## *In vitro* Studies of SP-D-Mediated Cellular Effects

Several *in vitro* studies have examined how SP-D modulates the function of a range of cell types (Table 1). This section focuses on recently published *in vitro* studies investigating SP-D-mediated cellular effects that might be relevant for the development of CVD.

**Table 1:** SP-D-mediated effects related to CVD.

**Cell type**	**Experiment**	**Outcome**	**References**
***IN VITRO*** **ANALYSIS**			
Human coronary SMCs	LPS-induced inflammation in coronary SMCs	SP-D suppresses the secretion of IL-8 in human coronary SMCs	(19)
PBMCs	PHA- or Con A-stimulated PBMCs treated with recombinant rat SP-D	SP-D inhibits T lymphocyte proliferation; both IL-2-dependent and -independent	(44)
PBMCs	PHA-stimulated PBMCs treated with rhSP-D	SP-D suppresses circulatory IL-6 and TNF-α levels	(45)
Plaque macrophages	Lipid-laden macrophages from atherosclerotic plaques	SP-D binding to OSCAR induced secretion of TNF-α from CCR2+ inflammatory monocytes	(46)
Monocytes and macrophages	Monocytes and macrophages expressing LAIR-1	SP-D binds LAIR-1 on monocytes and macrophages, thereby inhibiting ROS production	(47)
Pulmonary NK cells	Pulmonary NK cells expressing or not expressing NKp46	SP-D binds directly to pulmonary NK cells through the receptor NKp46, thereby inducing IFN-γ secretion	(48)
**Mouse model**	**Experiment**	**Outcome**	**References**
**ANALYSIS OF GENE-DEFICIENT MICE**			
SP-D/ApoE DKO mice	SP-D/ApoE DKO mice receiving proatherogenic diet	SP-D/ApoE DKO mice had reduced plaque lesion area, increased plasma cholesterol and weight, decreased plasma IL-6	(31)
SP-D/ApoE DKO mice	SP-D/ApoE DKO mice receiving proatherogenic diet with cholate	SP-D/ApoE DKO mice had reduced plaque lesion area, increased plasma triglycerides and HDL	(18)
SP-D KO mice	SP-D KO mice exposed to cigarette smoke	Control SP-D KO mice showed similar contractility in the coronary artery to that of cigarette smoking WT mice	(49)
SP-D KO mice	SP-D KO mice exposed to cigarette smoke	SP-D KO mice had aggravated airway inflammation and ceramide accumulation	(36)
SP-D KO mice	LPS-challenged SP-D KO mice	SP-D KO mice had increased testicular levels of immunosuppressive molecules, reduced levels of immune cell activation markers and reduced response to LPS in testis	(50)
**Patient cat**.	**Experiment**	**Outcome**	**References**
**CLINICAL PHENOTYPIC-ASSOCIATION STUDIES**			
Elderly twin population	689 elderly subjects, 13-year follow-up period.	Increased circulatory SP-D is associated with total mortality	(43)
Patients undergoing coronary angiography	806 patients, angiography happened between 1992 and 1995, follow-up in 2007.	Increased circulatory SP-D is associated with CVD morbidity and mortality	(42)
Patients undergoing maintenance hemodialysis	116 patients, cross-sectional study.	A positive association between circulatory SP-D level and carotid intima-media thickness and coronary artery calcification	(51)
Patients with heart failure	263 patients, 2.2-year follow-up period.	Circulatory SP-D is associated with a higher risk of heart transplantation, death, and worsened heart failure	(52)
PAD patients	364 patients, prospective study.	Circulatory SP-D is associated with more severe PAD and a higher prevalence of diabetes mellitus	(53)
Patients with subclinical carotid artery atherosclerosis	687 patients	No association between plasma SP-D and carotid artery intima-media thickness or subclinical atherosclerotic plaque development	(54)
Patients with chronic heart failure	89 patients and 17 healthy subjects	Circulatory SP-D levels are increased in patients with heart failure	(41)
Patients with non-massive and sub-massive pulmonary embolism	20 patients with non-massive, 20 patients with sub-massive and 20 healthy subjects	Circulatory SP-D correlated with the presence of sub-massive pulmonary embolism	(40)
**Patient cat**.	**Experiment**	**Outcome**	**References**
**CLINICAL GENETIC-ASSOCIATION STUDIES**			
206 healthy subjects	SNPs in the SP-D gene SFTPD	The SNP rs721917 (Met11Thr) affects the oligomeric structure of SP-D	(55)
2,711 type 2 diabetic patients or healthy subjects	SNPs in the SP-D gene SFTPD	The SNP rs721917 is associated with diabetes mellitus and insulin resistance	(56)
396 patients with subclinical atherosclerosis	SNPs in the SP-D gene SFTPD	The SNPs rs721917 and rs3088308 are both associated with decreased plasma SP-D; rs721917 is associated with carotid intima-media thickness	(54)

### Vascular Inflammation as an SP-D-Mediated Contributor to the Development of CVD

It is well established that chronic inflammation within the vessel wall is a strong contributor to the development of atherosclerosis. Chronic low levels of inflammation caused by pro-inflammatory molecules, such as lipopolysaccharide (LPS), has previously been implicated in the development of atherosclerosis (both in mice and humans) (57, 58). Although SP-D has been implicated in atherosclerosis, the exact mechanisms are very poorly understood.

**Figure 2:**
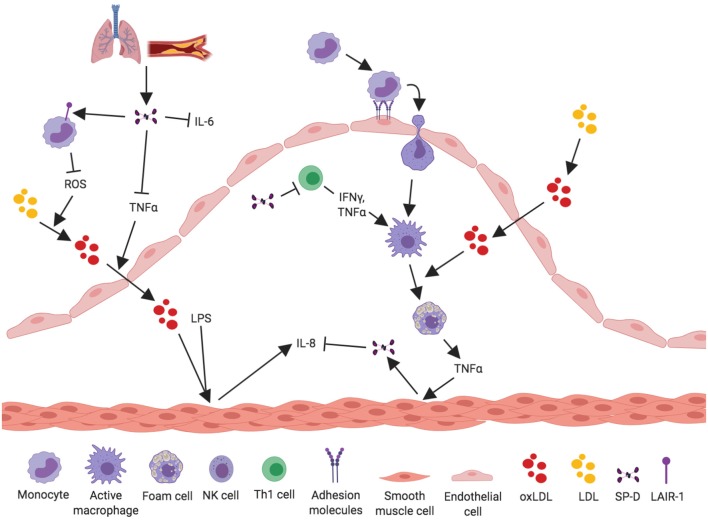
Proposed anti-inflammatory mechanisms for SP-D in the development of atherosclerosis. SP-D plays a dual role in the development of atherosclerosis. This figure aims to depict what we know about the anti-inflammatory properties of SP-D in the development of atherosclerosis. Circulatory SP-D originates from lung-spillover or directly from the atherosclerotic artery. SP-D binds its receptor LAIR-1 on PBMCs, leading to inhibition of ROS and thereby inhibition of the oxidation process of serum LDL. Circulatory SP-D also inhibits circulatory IL-6 and TNF-α and thus the transport of oxidized LDL from the artery lumen to the subendothelial space. SP-D has been shown to inhibit the proliferation of Th1 cells and their secretion of IFN-γ and TNF-α; this ultimately leads to inhibition of activated macrophages and foam cell formation. TNF-α secretion from NK cells and foam cells stimulates vascular smooth muscle cells (VSMC) to produce and secrete SP-D into the subendothelial space, where it again exerts its negative-feedback loop on NK cells. SP-D is able to inhibit IL-8 from LPS-stimulated VSMC, thus decreasing vascular inflammation.

It is recognized that SP-D/-A can modify the inflammatory response upon binding to LPS on the bacterial surface (59–61). Snyder et al. (19) examined the expression of SP-D in the human coronary artery SMCs and its ability to modify LPS-induced inflammation within these cells. After treatment of the human coronary artery SMCs with LPS, exogenously added SP-D had the ability to suppress the secretion of the pro-inflammatory cytokine IL-8 (Figure 2). Thus, SP-D is shown to have an anti-inflammatory role in SMCs when exposed to LPS and could possibly exert local protection against atherosclerosis in this way. A study by Liu et al. (62) examined the serum levels of the pro-inflammatory and proatherogenic cytokines IL-6 and TNF-α in septic wildtype and SP-D KO mice. This study showed that SP-D KO mice had significantly increased levels of both cytokines in serum. These results show that SP-D regulates not only local tissue inflammation but also systemic inflammation and, thus, possibly the propensity of atherosclerotic plaque formation.

### SP-D-Mediated Effects on Immune Cells

#### Peripheral Blood Mononuclear Cells

The cellular effects of SP-D on inflammatory cytokine levels are multifaceted. Borron et al. (44) investigated the effect of SP-D/-A on peripheral blood mononuclear cell (PBMC) proliferation and found that SP-D/-A directly inhibits T lymphocyte proliferation; both IL-2-dependent and -independent (Figure 2). A recent study by Pandit et al. (45) further examined how recombinant human (rh)SP-D affects phytohaemagglutinin (PHA)-stimulated PBMCs (45). The levels of two potent pro-inflammatory and pro-atherosclerotic cytokines, circulatory IL-6 and TNF-α, were significantly downregulated in rhSP-D treated PBMCs (Figure 2). Interestingly, this study observed no effect on IL-2 expression by rhSP-D (45). The identification of SP-D as a down-regulator of serum IL-6 and TNF-α is in agreement with the observations in Liu et al. (62).

Pro-atherosclerotic effects of IL-6 include vascular SMC proliferation (63, 64) and activation of endothelial cells (65, 66). However, the atheroprotective effects of IL-6 have also been reported, as IL-6 lowers plasma LDL by upregulating LDL receptor gene expression (67, 68). Clinical studies have shown that serum IL-6 is elevated in coronary artery calcification in patients with chronic kidney disease and might be a predictive biomarker of mortality risk, coronary artery disease, and inflammation related to CVD (69–71). Similar to the function of IL-6, TNF-α is also a pro-atherosclerotic cytokine and increases the development of atherosclerosis in several ways. Circulating TNF-α is associated with endothelial dysfunction and barrier disruption (72, 73), increased expression of adhesion molecules on endothelial cells (74), and vascular SMC proliferation (75).

#### Monocytes and Macrophages

**Figure 3:**
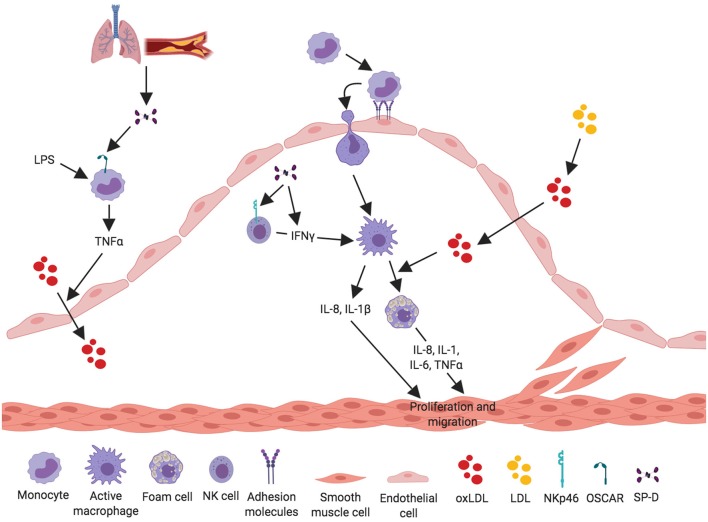
Proposed pro-inflammatory mechanisms for SP-D in the development of atherosclerosis. SP-D plays a dual role in the development of atherosclerosis. The figure aims to depict what we know about the pro-inflammatory properties of SP-D in the development of atherosclerosis. SP-D is actively contributing to the inflammatory process in the artery. SP-D stimulates LPS-induced TNF-α-secretion from CCR2+ inflammatory monocytes through binding via its receptor OSCAR. TNF-α stimulates translocation of oxidized LDL from the artery lumen to the subendothelial space and thus contributes to the atherosclerotic plaque formation. The pro-inflammatory role of SP-D is also mediated through NK cells, where SP-D binds the receptor NKp46 leading to secretion of IFN-γ, which activates macrophages. Activated macrophages secrete the cytokines IL-8 and IL-1 and are able to engulf the oxidized LDL, leading to foam cell formation and production of IL-8, IL-1, IL-6, and TNF-α. These cytokines stimulate and activate VSMC to proliferate and migrate to stabilize the growing atherosclerotic plaque.

Recently, a new receptor for SP-D was found on the cell surface of C-C chemokine receptor 2 (CCR2)+ inflammatory blood monocytes (46). The SP-D receptor osteoclast-associated receptor (OSCAR) was strongly expressed in lipid-laden macrophages localized in the tunica intima and in some of the macrophages in tunica media of clinical atherosclerosis plaques. Binding of SP-D to OSCAR induced secretion of TNF-α from the CCR2+ inflammatory monocytes infiltrating the atherosclerotic plaques (46) (Figure 3).

Olde Nordkamp et al. (47) found that SP-D binds the leukocyte-associated immunoglobulin-like receptor 1 (LAIR-1), a receptor expressed on most immune cells, including monocytes, and macrophages (76, 77) (Figure 2). SP-D inhibits reactive oxygen species (ROS) production through binding between SP-D collagen domains and LAIR-1 (47). ROS production is strongly associated with the development of atherosclerosis (78–80). A newer study by Yi et al. (81) demonstrated that silencing of LAIR-1 in human Tohoku Hospital Pediatrics (THP)-1 macrophages increases foam cell formation. This could be a possible link between SP-D and development of atherosclerosis, suggesting that binding between SP-D and LAIR-1 leads to a decreased foam cell formation, but this needs further examination.

These studies support a dual role for SP-D in the development of atherosclerosis. Signaling through OSCAR results in TNF-α secretion from monocytes that leads to a pro-inflammatory response, whereas signaling through LAIR-1 inhibits ROS formation but possibly induces foam cell formation.

#### NK Cells

A study by Ge et al. (48) showed that SP-D binds directly to pulmonary natural killer (NK) cells through the receptor NKp46 and thereby induces secretion of IFN-γ (Figure 3). This is the most specific receptor for NK cells and is expressed on the surface of all NK cell subsets (82, 83). It is possible that SP-D can bind and activate NK cells present in the artery, leading to secretion of IFN-γ, and thus stimulating an inflammatory response within the artery.

## *In vivo* Studies of SP-D in CVD

Two *in vivo* studies have been performed to investigate the role of SP-D in atherosclerosis (Table 1). A study by Hirano et al. examined the function of SP-D in the development of atherosclerosis in ApoE (KO) mice (31). A similar study was previously performed by Sorensen et al. in SP-D KO mice receiving a proatherogenic diet containing a low percentage of cholate (18).

The function of SP-D has also been examined in relation to tobacco smoking, as tobacco is a major contributor to CVD. Haanes et al. (49), examined the contractile changes in the vasculature after 3-months sidestream smoking in wildtype and SP-D KO mice.

### Plaques Size and Composition

Hirano et al. (31) assessed the development of atherosclerosis in ApoE KO and SP-D/ApoE double KO (DKO) mice. Atherosclerotic plaque size was measured in the brachiocephalic arteries and aortic root where plaque formation often is seen. The DKO mice exhibited significantly reduced plaque size in both the brachiocephalic arteries (37% reduction) and aortic root (25% reduction) compared to ApoE KO mice (31). This result is supported by Sorensen et al. who examined the function of SP-D in relation to atherosclerosis in mice fed an atherogenic diet (18). These authors found that SP-D KO mice had significantly smaller lesion areas only consisting of foam cells, whereas WT mice had larger mature atherosclerotic plaques with an extracellular matrix containing lipid and collagen (18).

Furthermore, Hirano et al. revealed that the plaques in SP-D/ApoE DKO mice harbored significantly fewer macrophages in each plaque (46% reduction) and had more SMC coverage of the luminal surface of the plaque compared to ApoE KO mice. SMC coverage of the luminal surface of the plaque is associated with a more stable plaque phenotype in humans (84, 85). Based on these results it seems that SP-D has a function in the attraction of macrophages and thereby initiation of early inflammation, which ultimately leads to the development of more severe atherosclerosis. Furthermore, it seems as if SP-D has a function in changing the stability of the plaque, leading to a more unstable plaque formation and a higher risk of plaque rupture.

### Lipoprotein Profile

The smaller plaque areas observed in SP-D KO and SP-D/ApoE DKO mice were hypothesized to be caused by an SP-D-mediated change in the lipoprotein profile, leading to a lower degree of lipids and cholesterol in the blood. Both Hirano et al. and Sorensen et al. detected a significant change in the lipoprotein profile of the SP-D KO and SP-D/ApoE DKO mice (18, 31). Surprisingly, SP-D/ApoE DKO mice had a 39% higher fasting plasma level of cholesterol and significantly higher levels of chylomicron cholesterol, very-low-density lipoprotein cholesterol (VLDL-C), as well as high-density lipoprotein cholesterol (HDL-C) (31). In addition to disturbed blood lipids, SP-D/ApoE DKO mice had elevated body weight and adipose tissue mass, as well as increased fasting plasma insulin, glucose, leptin, and adiponectin compared to ApoE KO mice (31). Sorensen et al. found that SP-D KO mice had significantly higher levels of plasma HDL-C (18%) and triglycerides (27%) but similar levels of LDL and total cholesterol compared to WT mice (18). Sorensen et al. (18) examined the effect of intravenous administration of rhSP-D on plasma lipid concentrations. Administration of rhSP-D to SP-D/ApoE DKO mice decreased the serum concentration of HDL-C, LDL-C, and total cholesterol. This SP-D-mediated regulation of multiple lipid fractions indicates that a general factor in lipid metabolism or transport is affected by SP-D.

It is well-known that obesity, insulin resistance, increased fasting glucose, and increased levels of plasma VLDL, LDL, and cholesterol are associated with the development of atherosclerosis and CVD (86–90). However, the SP-D mediated mechanisms modulating these metabolic outcomes and how they are interlinked with CVD remain unknown.

### Inflammation *in vivo*

To investigate if the effect of SP-D on plaque size was caused by a change in inflammatory markers, Hirano et al. (31) and Sorensen et al. (18) evaluated the level of cytokines in plasma. Strikingly, SP-D/ApoE DKO mice had significantly lower plasma level of IL-6 compared to ApoE KO mice (31). It has previously been shown that injection of IL-6 in ApoE KO mice increases the levels of pro-inflammatory cytokines and lesion size and that the use of IL-6 lentivirus led to the destabilization of plaques (15, 16). The decreased plasma IL-6 level could, therefore, be partly responsible for the attenuation of atherosclerosis in SP-D/ApoE DKO mice, despite the elevated body weight and plasma lipoproteins, which are known risk factors for the development of CVD (86–90). However, this is not in accordance with an *in vitro* study by Liu et al. (62), who found that serum levels of IL-6 and TNF-α were increased in septic SP-D KO mice compared to wildtype. This discrepancy can be explained by a change in the function of SP-D during sepsis—where it possibly functions as a neutralizer of the sepsis-inducing agent—compared to its function at basal state.

Furthermore, SP-D/ApoE DKO mice had significantly reduced absolute blood monocyte (46% reduction) and neutrophil counts (49% reduction), as well as a 46% reduction in spleen weight (relative to body weight) compared to ApoE KO mice (31). The spleen is a major reservoir of both monocytes and neutrophils, and its reduced weight can explain the decreased monocyte and neutrophil counts. Sorensen et al. found that SP-D KO mice had a significantly lower level of plasma TNF-α (18), which could contribute to the lower atherosclerosis propensity, similar to the IL-6 regulation mentioned above. TNF-α is associated with an increased risk of CV disease in clinical studies (91), and TNF-α/ApoE DKO mice had reduced lesion areas (50%) with a decreased number of foam cells in the vessel wall (92).

### SP-D-Mediated Responses to Smoking

Tobacco smoking is a well-studied cause of CVD (93). A study by Haanes et al. (49), examined the contractile changes in the vasculature after 3-months sidestream smoking in wildtype and SP-D KO mice. Haanes et al. (49) studied the vasocontractile endothelin-1 receptor and the uridine diphosphate receptor P2Y6 in the pulmonary artery left anterior descending coronary artery and the basilar artery. Endothelin-1 sensitivity was not affected by SP-D. When stimulating the P2Y6 receptor in the coronary artery, there was a significantly decreased contraction after cigarette smoking in the wildtype mice compared to control wild-type mice. This significant decrease in contraction was not observed after smoking in the SP-D KO mice compared to control SP-D KO mice. Thus, the control SP-D KO mice had similar UDP-induced contractility in the coronary artery to those of cigarette smoking WT mice.

Although the SP-D mediated mechanisms were not investigated, the observation suggests that the SP-D-deficient artery is more vulnerable to inflammatory stimuli, such as tobacco smoking.

Pilecki et al. (36) recently performed a study combining observations of SP-D immunostaining in resected lung tissue from smoking patients with both *in vitro* and *in vivo* experiments. These authors found an increased expression of SP-D in lungs exposed to cigarette smoke, that SP-D deficiency aggravated airway inflammation and ceramide accumulation in mice exposed to cigarette smoke, and that treatment with rhSP-D ameliorated cigarette smoke-induced inflammation and epithelial apoptosis in both mice and alveolar (A)459 cells. This indicates that SP-D takes part in the regulation of ceramide synthesis, which has also been implicated in atherosclerosis. Ceramides promote the secretion of IL-6 and C-reactive protein (94), increases ROS formation (95), promotes transcytosis of oxLDL across endothelial cells (96), and increases monocyte adhesion (97), thereby promoting inflammation and atherosclerosis (98, 99).

## SP-D Variation in Clinical Studies

It has long been known that chronic lung disease is associated with increased risk of CVD and total mortality. Independent studies have suggested that SP-D might function as a blood biomarker of smoke-induced lung injury and chronic obstructive pulmonary disease (COPD) (Table 1) (100–103). COPD is an independent risk factor for coronary artery disease, and COPD patients are at increased risk of death due to CVD (104, 105).

### Association Between Circulatory SP-D, Total Mortality, and CVD

A recent elderly twin population study by Wulf-Johansson et al. (43) examined the correlation between circulating SP-D levels with total mortality. The twin with highest-circulating SP-D level had a significantly increased risk of dying before the co-twin during the study follow-up period. Adjusting for smoking did not change this result. This observation is supported by Hill et al. (42), who examined the correlation between circulating SP-D and the risk of cardiovascular morbidity and mortality. The study found that increased serum SP-D was associated with total mortality in patients with documented coronary artery disease also when adjusting for well-established risk factors such as smoking, age, sex, plasma cholesterol, and plasma IL-6 levels. The relationship between SP-D and CVD was further supported by Hu et al. (51), showing a positive association between circulatory SP-D level and carotid intima-media thickness and coronary artery calcification.

A new study by Brankovic et al. (52) examined the potential of SP-D as a biomarker in patients with chronic heart failure. Brankovic et al. (52) reported that SP-D was associated with a worsened clinical outcome (worsened heart failure, heart transplantation, death to CVD) independently of the patient's clinical profile and pharmacological treatment during the follow-up period. However, SP-D did not remain as a significant biomarker after adjustment for time-varying cardiac biomarkers, including N-terminal pro-brain natriuretic peptide and high-sensitivity cardiac troponin T (52). The study mainly based its results on a population with a less severe heart failure (74% was in NYHA class 1 or 2), which might have contributed to the failure to show a robust association between SP-D and heart failure, since lung damage is more pronounced in the more advanced stages of chronic heart failure (41). Another recent study by Otaki et al. (53) examined the association between SP-D and the outcome of peripheral artery disease (PAD) patients. PAD is an occlusive disease in the lower limb arteries and is a well-known risk factor for the development of CVD and death. Patients with a high circulatory SP-D level (≥ 100 ng/mL) had more severe PAD determined using the Fontaine class according to TASC II guidelines (53). Patients with high SP-D also had a higher prevalence of diabetes mellitus and tibial or peroneal artery stenosis/occlusion compared to patients with low SP-D. Furthermore, the study found an association of SP-D levels with endpoints, such as cardiovascular death, heart failure, and leg amputation. The study concluded that circulating SP-D could be a potential therapeutic target and also be useful as a biomarker for tracking atherosclerotic health status in PAD patients.

### Single Nucleotide Polymorphisms in the SP-D Gene Related to CVDs

Leth-Larsen et al. (55) and Pueyo et al. (56) examined single nucleotide polymorphisms (SNPs) in the SP-D gene *SFTPD* and their relationships to SP-D oligomerization and development of diseases (55, 56). Leth-Larsen et al. (55) showed that a common SNP, rs721917, resulting in either threonine or methionine at position 11 in the mature protein (Met11Thr), affects the oligomeric structure of the SP-D molecule. Interestingly, this SNP was also found to be associated with type 2 diabetes mellitus and insulin resistance independently of circulatory SP-D levels (56). A recent study by Sorensen et al. (54) examined the relationship between plasma SP-D and subclinical carotid artery atherosclerosis. The study found no association between plasma SP-D and carotid artery intima-media thickness or subclinical atherosclerotic plaque development. Sorensen et al. (54) further examined two SNPs in the SP-D gene *SFTPD*: rs721917 and rs3088308. Both SNPs were significantly associated with a decreased plasma SP-D level independently of patient smoking status. Moreover, rs721917 was significantly associated with carotid intima-media thickness, whereas rs3088308 was significantly associated with the presence of plaques—both as a smoking-dependent effect of the SNPs (54). Thus, the effects of SP-D are likely caused by an interplay between structural SP-D variations and tobacco smoking.

## Discussion

SP-D can undergo several post-translational modifications leading to changes in both structure and function (106, 107). The most common isoform is the dodecameric structure, which is thought to have anti-inflammatory properties (29, 106). However, in an inflammatory environment, the dodecameric form can change to a trimeric or monomeric isoform that is believed to have pro-inflammatory properties (106) (Figure 1). This could explain the ambiguous role of SP-D as both an anti- and pro-inflammatory stimulator in the development of CVD and in the absence of microbial binding. Most studies do not examine which SP-D isoform exerts the observed effect, and therefore the relationship between SP-D isoforms and risk of CVD and all-cause mortality is unknown. It is also relevant to examine the correlation between not only total circulatory SP-D and CVD but also between SP-D polymorphisms and CVD; Met11Thr is especially known to affect the function of SP-D. Also, many studies do not report the content of SP-D in BALF or other measurements of lung disease and thus do not consider where the circulatory SP-D originates from. In a recent study by Sarashina-Kida et al. (108), it was shown that SP-D could also originate from the gallbladder, where it had an anti-inflammatory role in a dextran sulfate sodium (DSS)-induced murine colitis. However, Nexoe et al. (109) performed a similar study and could not replicate the anti-inflammatory role of SP-D in the DSS-model. Regardless, the conclusion is that SP-D not only originates from the lung but can be secreted by various sources, such as the arteries and gallbladder. Where the SP-D originates from is important since previous studies have suggested that SP-D might be upregulated in non-pulmonary organs during inflammation. Future studies are needed to explore which tissues and cell types secrete SP-D.

When interpreting results from *in vivo* studies examining SP-D KO mice, it is important to keep in mind that these data can sometimes be misleading. Deficiency of an immunoregulatory protein, such as SP-D, might lead to a compensatory increase in other immunoregulatory proteins. This compensatory mechanism might lead to a different outcome than expected. Rokade et al. (50), examined the function of SP-D in testicular immune privilege and sperm function in LPS-challenged SP-D KO mice. These authors found that endogenous absence of SP-D resulted in significantly increased testicular levels of immunosuppressive molecules and reduced levels of immune cell activation markers. The change in immunoregulative moieties led to a reduced response to LPS in testis and could be a compensatory mechanism to restore the immune dysregulation caused by SP-D KO to preserve fertility. In a similar way, SP-D KO in murine CVD models might promote compensatory mechanisms, resulting in reduced plaque areas and immune cell counts.

Studies examining the function of SP-D have previously been focused on its association with pulmonary diseases, especially COPD. It is important to elucidate the role of SP-D in other pathologies, and its recent association with CVD supports the possibility of SP-D as a potential therapeutic target. The effect of exogenously added SP-D in atherosclerotic mouse models needs further validation but results from basic cardiovascular studies suggest systemic side effects, such as disturbance of lipoprotein levels and body fat distribution. These studies imply that treatment with exogenously added SP-D, when possible, should be locally administered instead of systemically. The conformation-dependent pro- or anti-inflammatory role of SP-D should be further elucidated, as blockage or stimulation of certain isoforms can change the SP-D-mediated treatment effect. Besides being a target for CVD treatment, SP-D has shown promise as a new potential biomarker for the severity and clinical outcome of CVD. Studies have shown significant association between circulatory SP-D levels and development of atherosclerosis and heart failure. Circulatory SP-D might reflect the level of atherosclerosis or the risk of cardiovascular-related death and thus the clinical outcome. It is important to continue examining the association between SP-D and CVD to fully elucidate the potential of SP-D as a treatment target and/or CVD biomarker.

## Author Contributions

KC was the main author of the manuscript under the guidance of professor GS. AN supported the writing of the manuscript.

### Conflict of Interest

The authors declare that the research was conducted in the absence of any commercial or financial relationships that could be construed as a potential conflict of interest.
